# Mental Health Management of English Teachers in English Teaching Under the COVID-19 Era

**DOI:** 10.3389/fpsyg.2022.916886

**Published:** 2022-06-09

**Authors:** Yiling Ding, Tianhua Wang

**Affiliations:** ^1^Heilongjiang University, Harbin, China; ^2^Harbin Normal University, Harbin, China

**Keywords:** COVID-19, English teachers, mental health, graph convolutional networks, bipartite

## Abstract

**Background:**

The COVID-19 pandemic has brought new challenges and attention to the mental health of all social groups, making mental health increasingly necessary and important. However, people only focus on the mental health of undergraduates, and the mental health of teachers has not received much attention from society. College teachers are the backbone of the teachers' group, and their mental health not only affects the teaching quality and research level but also plays an important role in the mental health and personality development of undergraduates.

**Method:**

During the COVID-19 pandemic, online teaching is a major challenge for college teachers, especially English teachers. To this end, this article proposes a bipartite graph convolutional network (BGCN) model based on the psychological test questionnaire and its structural characteristics for the recognition of the mental health crisis.

**Results:**

Experimental results show that the proposed BGCN model is superior to neural network algorithms and other machine learning algorithms in accuracy, precision, F1, and recall and can be well used for the mental health management of English teachers in the era of COVID-19.

## Introduction

Teachers' mental health is a cross-concept between social psychology and pedagogy (Tsukawaki and Imura, 2022). It not only has the basic characteristics of general individual mental health but also the traits that are defined by the teacher's profession. The psychological diathesis of teachers mainly refers to the essential characteristics of the psychological process and individual psychological characteristics of teachers, which determine their teaching effect and influence the students' physical and mental development in educational and teaching activities (Gholamitooranposhti, 2012; Shah and Kumar, 2012). Teachers' mental health is closely related to teachers' diathesis, among which, mental health is the most basic psychological diathesis of teachers. It is easy for a teacher with good psychological diathesis to achieve the goal of mental health (Kovess-Masfety et al., 2007; Yang et al., 2019).

College English teachers' mental health is the prerequisite for teachers to complete normal English teaching. It is not only a guarantee for teachers to do well in English teaching and improve teaching quality and efficiency but also the basis for undergraduates' mental health education (Rothi et al., 2008). Only teachers with mental health can cultivate students with mental health. Teachers' mental health directly affects undergraduates' mastery of English, their ability to use English in practice, and even their sociality and personality. College English teachers need to broaden their professional knowledge and improve their scientific and technical development. Therefore, with the progress of society, college English teachers can only become qualified English educators if they fully understand the role they play and have good psychological quality and adaptability (Hofmann et al., 2022).

The mental health of college English teachers is an extremely complex and dynamic process (Kush et al., 2021), which is influenced by many factors. The specific manifestations are as follows.

1. Pressure of updating English knowledge: As the imparters of English knowledge, English teachers need to constantly explore the latest and cutting-edge English knowledge to meet their own development and the growing needs of students' English knowledge, so as not to be eliminated by the times (Dai, 2021). Therefore, college English teachers are facing great pressure and sense of crisis in knowledge renewal.
2. Social and college environment: At present, society and families have high expectations for college English education. Compared with the continuous development of social needs and teaching reform objectives, the teaching level of English teachers is relatively lagging behind, and there is a certain gap with social expectations, leading to the anxiety of college English teachers (Li, 2021). In addition, teachers are also under great pressure in terms of professional title evaluation and salary.
3. Double pressure of research and teaching: As college English teachers, on the one hand, they should be responsible for teaching English knowledge to students, improving students' English application level, and completing daily teaching tasks with high quality. At the same time, the current educational situation also requires college English teachers to make great efforts in research (Borg and Liu, 2013; Zheng, 2015). Therefore, college English teachers have to spend a lot of time and energy consulting literature or conducting practical investigations, which will lead to college English teachers' physical and mental fatigue and have an adverse impact on the mental health of teachers.
4. Influence of college English teaching evaluation mechanism: At present, the purpose of teacher evaluation in most colleges is only to improve the work and improve the quality of teaching, and the long-term development of teachers is less involved. It is a common problem that college English teachers lack scientific and systematic teaching theories and weak research abilities. However, there are few opportunities for college English teachers to go out for study, training, or further study; their professional development and improvement of their own quality are often carried out only through a single training; and the effect is not obvious. Meanwhile, the evaluation of college English teachers is mainly based on undergraduates' grades to evaluate their teaching effects (Bacher-Hicks et al., 2019; Rodl et al., 2020). This often leads to a large number of college English teachers focusing on “exam-oriented” teaching, whereas ignoring relevant research and their own career development.

Some studies have investigated the mental health status of college teachers in China under public health emergencies, and the results show that fear accounts for most of the mental health of college teachers, which is consistent with the psychological responses of the public, undergraduates, and medical staff. Although the educational quality of college teachers is generally high, they are not different from the general population due to the high infectivity of COVID-19, the complexity of transmission routes, and the lack of specific treatment drugs, so they are prone to fear and worry (Aperribai et al., 2020). It can be seen that in the face of public health emergencies, college teachers' fear responses also need more attention and intervention. During the pandemic, teachers in colleges have different levels of mental health problems, which are affected by many factors. Colleges should pay more attention to teachers who are divorced, isolated, or with chronic diseases.

The mental health level of English teachers has a great influence on undergraduates in English teaching. The mental health of undergraduates is inseparable from that of teachers. Teachers with a high level of mental health can weaken students' negative emotions in learning, teach undergraduates to solve all kinds of problems in life with an optimistic attitude, infect undergraduates with their own emotions, and bring positive energy to undergraduates' learning and life. Teachers' optimistic life attitude and fair and just way of doing things will have a positive impact on undergraduates' mental health, which is conducive to undergraduates' study and life. At the same time, teachers' emotions can easily affect undergraduates. Therefore, English teachers should control their emotions in teaching. Teachers' negative emotions will make undergraduates depressed in learning, reduce their enthusiasm for learning, and affect their learning efficiency.

To sum up, from the perspective of the current working and living conditions of college English teachers, under the double pressure of teaching and research under COVID-19, teachers' mental health is not optimistic, which objectively affects not only their own development but also their teaching attitude, teaching philosophy, and teaching behavior. Good teachers' psychological diathesis not only has the power to motivate teachers to work hard and give full play to their creative ability but also has a positive impact on the formation and development of students' personality, and the influence mechanism of teaching is irreplaceable by any other educational means. A psychological test questionnaire is a widely used tool for mental health detection (Yin et al., 2021). However, there are many deficiencies in the traditional calculation method of using a scale directly to recognize individuals in a psychological crisis, leading to a high false-positive rate and false-negative rate. In this article, an individual recognition method for the psychological crisis in teachers based on graph convolutional networks (GCNs) is proposed to make up for the deficiency of traditional recognition methods.

The contributions of this article mainly include: (i) a bipartite GCN (BGCN) model based on the psychological test questionnaire, and its structural characteristics is proposed for the recognition of the mental health crisis of English teachers; and (ii) we analyze the measures to promote the mental health of college English teachers and study the implementation path to improve the mental health level of English teachers.

The rest of the article is structured as follows. In Section “Mental Health Crisis Recognition Model for English Teachers”, the mental health crisis recognition model for English teachers is proposed. Experimental results are reported in Section Experiments and Results Analysis, and the measures for the mental health of college English teachers' promotion are also analyzed in Section Experiments and Results Analysis. Section Conclusions gives the conclusion of this article.

## Mental Health Crisis Recognition Model for English Teachers

To improve the recognition accuracy of the model and improve the generalization of the model, the trained model can be used to recognize teachers who do not appear in the networks. In this study, teachers and the questions in the psychological test questionnaires are used as nodes at the same time, and two kinds of nodes with different properties are connected to form a bipartite graph. A teachers' mental health crisis recognition method based on BGCN is proposed. Since teachers and questionnaires can be separated and reorganized, it is expected to improve the generalization and practicability of the model by reducing the coupling between teachers' nodes and problem nodes in the graph (Jin et al., 2021).

### Graph Construction

Let *Q*, *T*, *E*, and *W* be the question node set of the psychological test questionnaires, the teacher node set, the set of edges in the bipartite graph, and the weight set of corresponding edges, respectively, thus forming the bipartite graph *G*(*Q, T, E, W*).

#### Calculation of Weight Matrix

The teacher node *T* connects an edge with each psychological test questionnaire question node *Q*, and the edge weight *W* is set according to the answers to psychological test questionnaire questions. There are two kinds of answer options in the psychological test questionnaire. One is true or false, where the answer is yes or no. The other one is a clicker question, where the answer options are used to indicate the degree of a certain situation. Most of these psychological test questions are divided into five levels of options, including strongly agree, agree, neutral, disagree, and strongly disagree.

Converting the answers in both cases to numbers: For the true or false questions, the weight is set to 0 or 1, in which 0 means that the teacher answers no to the question, and 1 means that the teacher answers yes to the question. For the clicker questions, assuming that there are *n* options, the teacher's options are represented as integers between 0 and *n*−1 according to five levels of options.

The answer matrix *A* formed by the teacher node question and the psychological test questionnaire question can be obtained by converting the answers of all the teacher node questions.

#### Weight Matrix Preprocessing

In experiments, the teacher–questionnaire bipartite graph is not a directly used transformed weight matrix. At first, it is necessary to standardize the weight values of each specific problem and obtain the normalized weight matrix A^*^, so that the weight values of each problem are mapped to the same distribution, reducing the influence of differences between different problems.

#### Graph Generation

In this study, the complete bipartite graph can be constructed in two ways: an undirected graph and directed graph. In the setting of the undirected graph, the interaction between the specific question node and the specific teacher node will be strengthened rapidly, which is conducive to the classification of the psychological crisis of teachers. Thus, this article studies the model based on the undirected graph. For the undirected graph *G*_*undi*_(*Q, T, E, W*), the set of edge weight *W* directly corresponds to the value in a normalized weight matrix A^*^.

#### Principle of Graph Construction

The model assumes that the hidden features of teacher nodes will be affected by the question nodes in the psychological test questionnaire, and its influence is related to the answer matrix *A* of teacher nodes. It is assumed that the influence of problem nodes on teacher nodes is the same as that of teacher nodes on problem nodes. Under this assumption, the model uses the undirected graph *G*_*undi*_(*Q, T, E, W*).

### Model Introduction

The model assumes that the hidden features of teacher nodes can be calculated according to the characteristics of the questions in the psychological test questionnaire, and the influence of each question on teacher characteristics is related to the teacher's answer to each question (the influence of question node features of answer *a* on teacher node features is *Infl*(*a*)). The model also assumes that the hidden features of question nodes can be calculated according to the features of teacher nodes. Assuming that the influence of the teacher node on the question node is the same as that of the question node on the teacher node, both of which are *Infl*(*a*), an undirected bipartite graph is formed.

The forward propagation process and calculation details of the model are as follows.

#### Initial Features and Models Definition

The matrix *Q* is defined as the eigenmatrix of the question, *Q* = *I*_*k*_, and *I*_*k*_ is the *k*-dimensional identity matrix. *T* is defined as the eigenmatrix for teachers, and *T* is the zero matrix with k^*^n dimensionality. Model eigenmatrix is *P* = [*Q*∥*T*], and ∥ represents transverse connection operation. The weighted adjacency matrix of the complete bipartite graph *G* used by the model is defined as *A*, so the BGCN model can be represented by the function *f*(*P, A*).

#### Graph Convolutional Layer

**Figure 1:**
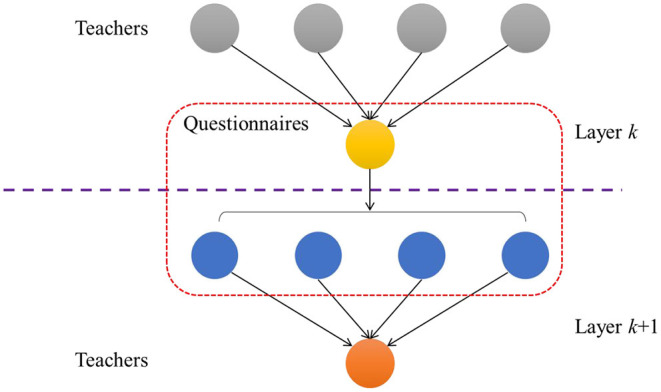
A two-layer graph convolution process.

Unlike the general convolution process, the bipartite graph convolution needs two times of convolution so that the information of the teacher node can return to the teacher node again through the question node. Therefore, even convolutions are required to ensure that the representation of teacher nodes is appropriate. As shown in Figure 1, on the convolution of the *k*th layer graph, the teacher node first passes the feature information to the question node. At the *k*+1th layer, the question node is transferred back to the teacher node.

#### Dropout Layer

In the training of machine learning models, if the model has more parameters and fewer training samples, then the trained model is prone to overfitting, which is manifested in the smaller cost function and higher prediction accuracy of the model on the training set, but the larger cost function and lower prediction accuracy on the test set. Such a phenomenon may occur in all kinds of machine learning models, especially when training neural networks.

To solve the problem of overfitting, the model integration method is generally adopted, that is, training multiple models to combine. However, model integration requires training and testing of multiple models, which can be time-consuming. Deep learning takes another approach to alleviate the overfitting problem by adding a dropout layer after each convolutional layer.

To prevent overfitting, the model performance can be improved by blocking the information transfer of hidden layer neuron nodes. That is, each neuron has a certain probability of being dropped out, so that its information cannot be conveyed to the next layer. After the dropout layer is introduced, each neuron node has a certain probability of not being activated, and the weight update of the model is no longer dependent on the joint action of fixed neuron nodes, which increases the robustness of the model (Liu et al., 2020).

#### Output Layer

After the question node feature and teacher node feature are convolved by the graph with an even number of layers, each node gets a new hidden feature. This feature calculates the likelihood of the teacher node belonging to a normal individual or crisis individual through the fully connected layer and then converts it into the probability of the teacher belonging to two categories through the Softmax function (Luo et al., 2020). Generally, the category with the highest probability of model output is the category of teachers. This model uses the negative logarithm likelihood function as a cost function. When solving the minimization cost function, it is equivalent to solving the maximum likelihood function. A logarithm is used to convert multiplication of probability to addition, and negative operation is used to convert maximization to minimization.

## Experiments and Results Analysis

### Dataset

This article mainly uses the Beck Depression Inventory (BDI) (Williams et al., 2021) and the Eysenck Personality Questionnaire (EPQ) (Colledani et al., 2018) as datasets. BDI is a measure of depression, and the whole scale consists of 21 groups of items, each of which has four statements. While EPQ is a questionnaire with 85 items that evaluates personality traits such as extroversion and emotional stability, including four parts: extraversion scale, neuroticism scale, psychoticism scale, and lie scale. Therefore, the dataset used in this article, that is, the psychological test questionnaire, includes BDI and EPQ, with a total of 106 items. To explore the relationship between the psychological test questionnaire and the psychological crisis of teachers, *t*-distributed stochastic neighbor embedding was used to reduce the dimensions of the psychological test questionnaire data, and it was found that there was no obvious distribution of the psychological crisis of teachers.

### Setup

Due to the limited number of English teachers, this study expands the scope of psychological test questionnaires. In total, 2,714 teachers from Heilongjiang University and 1,768 teachers from Harbin Normal University were selected to complete the psychological test questionnaires. We encrypted the data before conducting our research.

In total, 265 positive samples and 1,000 negative samples were selected to form the experimental sample set. In total, 20% of the samples, 53 positive samples, and 200 negative samples, were randomly selected as the test set, and the data were used as the training set to learn the parameters of the BGCN model. During the model testing phase, the teachers in the test set were used to construct a new bipartite graph, but the parameters learned by the BGCN model remained unchanged. The model used in the experiment was the two-layer graph convolutional neural network (CNN) model. The dropout layer was added between the convolutional layers, the dropout probability was set to 0.5, and the feature dimension of the hidden layer was set to 80 and 30, respectively. Adam optimization function was used, the learning rate was set to 1e−2, and the negative log likelihood function was used as the cost function.

### Comparison With Neural Network Models

**Figure 2:**
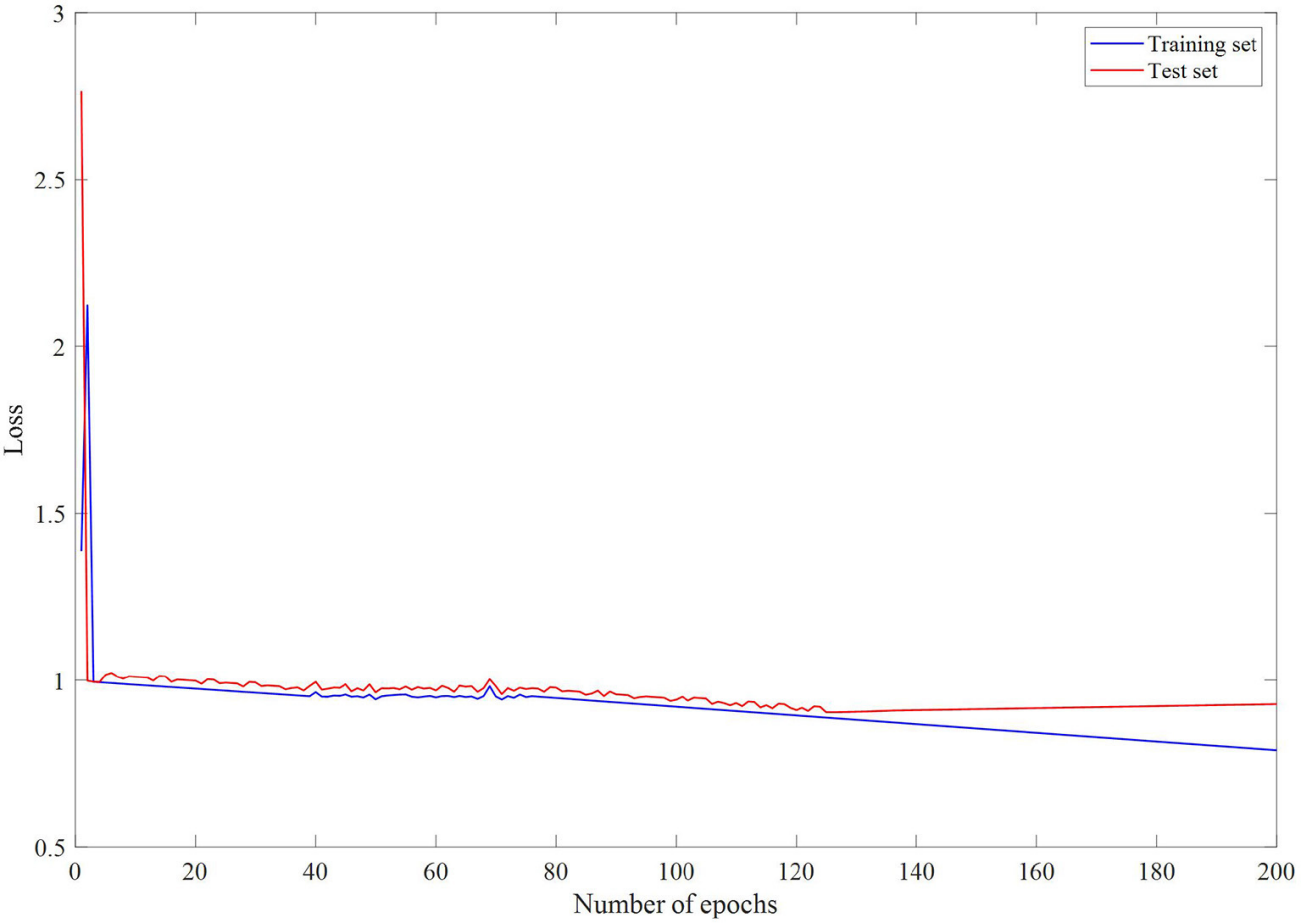
Loss changes with epochs.

**Figure 3:**
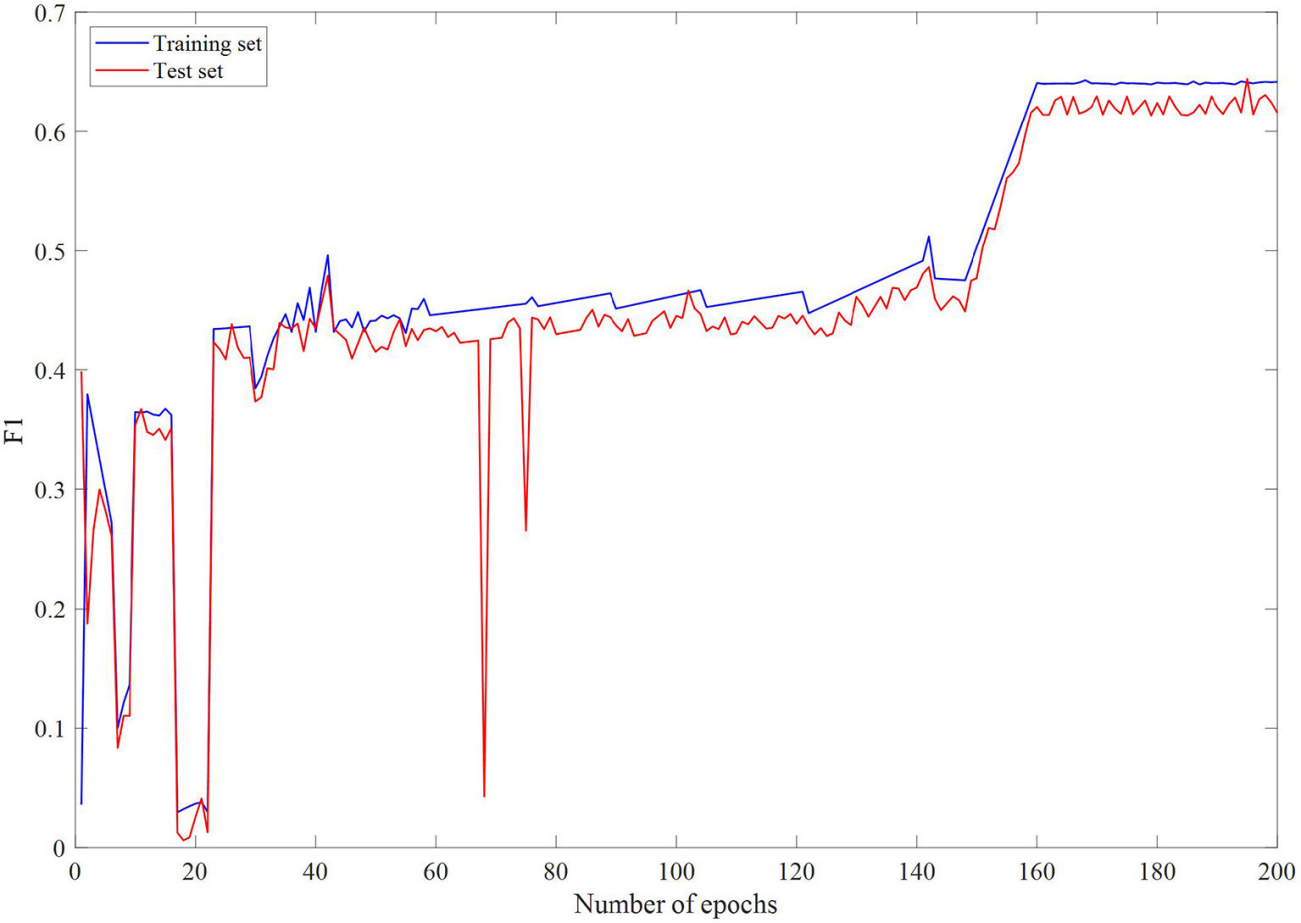
F1 changes with epochs.

**Figure 4:**
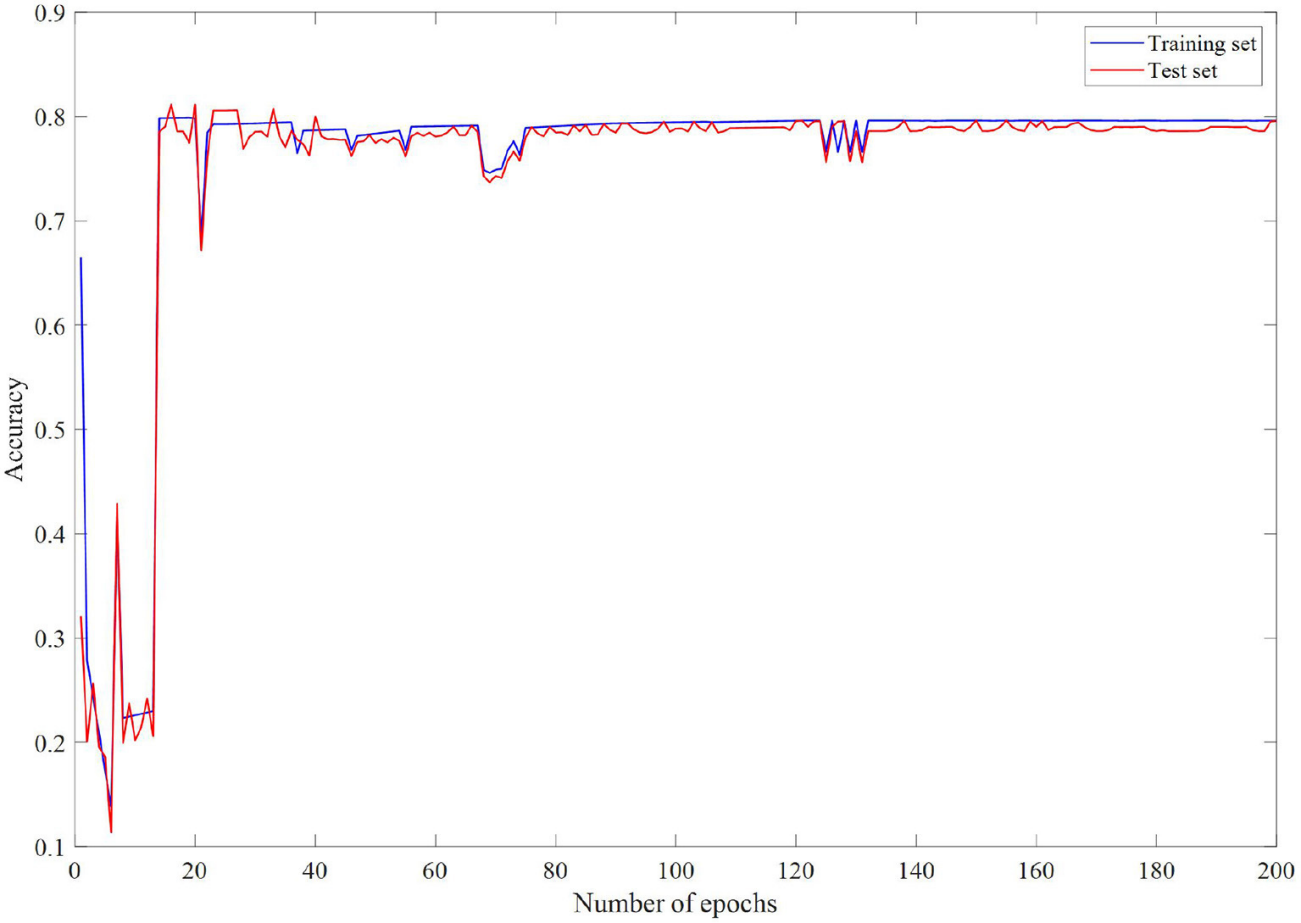
Accuracy changes with epochs.

In the case of undirected graphs, the teacher node is equally influenced by the question node, and the question node is equally affected by the teacher node. This means that if the features of a question have a great influence on the features of the teacher node, then the features of the teacher node also have a great influence on the features of the question. Figures 2–4 are loss changes, F1 changes, and accuracy changes, respectively. It can be seen from Figures 3, 4 that in the early epoch of undirected graph training, the model may mark a large number of teachers as mentally healthy teachers, but after a while, the model tends to be stable and gradually converges.

Table 1 compares the model performance of BGCN, GCN, CNN, and artificial neural network (ANN) (Maruyama et al., 2019). It can be seen from Table 1 that the ANN model only uses the teacher node to construct the teacher psychological similarity graph, and its model performance is significantly lower than the other three models, whereas BGCN uses the teacher–question bipartite graph with more information. Specifically, BGCN improved accuracy by 14.97% and F1 by 43.59% compared with ANN. The accuracy of BGCN is nearly twice that of the ANN model. Moreover, the GCN model cannot classify teacher nodes that have never been seen before, so it is not suitable for use in practical scenarios, whereas the BGCN model does not have this problem. For the newly added teacher node, it only needs to convert its options into two-layer graph convolution and access the trained network model to recognize it.

**Table 1:** Comparison of evaluation metrics with different neural network models.

**Method**	**Accuracy**	**Precision**	**Recall**	**F1**
ANN	0.7869	0.4562	0.5846	0.5735
CNN	0.8532	0.7648	0.6847	0.6648
GCN	0.8764	0.7418	0.7038	0.7157
BGCN	0.9047	0.8913	0.6472	0.8235

It is worth noting that some teachers in this study are labeled as individuals in psychological crisis by the model, but it is not clear whether the teachers suffer has psychological diseases. In fact, these teachers recognized by the model as individuals in a mental health crisis may have certain risks, and may even be ill without being diagnosed, so the college should give care and attention to such teachers.

### Comparison With Traditional Machine Learning Algorithms

In comparison with traditional machine learning algorithms, such as logistic regression (LR), support vector machine (SVM), and CNN, L2 regularization was used for LR, cross-entropy was used as the cost function, random-gradient descent was used to update model parameters, and the learning rate was set as 1e−3. The penalty coefficient of SVM is set as 1, radial basis function is taken as the kernel function, and the hyperparameter is set as 0.007. CNN was set as 4 layers, the number of neurons of the first hidden layer was set as 80, and the number of neurons of the second hidden layer was set as 30. The cost function is cross-entropy, the model parameters are optimized by stochastic-gradient descent, and the learning rate is 1e−3. Table 2 shows the experimental results.

**Table 2:** Comparison of evaluation metrics with different machine learning algorithms.

**Method**	**Accuracy**	**Precision**	**Recall**	**F1**
LR	0.7591	0.6125	0.5590	0.7644
CNN	0.8512	0.6678	0.4837	0.7719
SVM	0.8867	0.8215	0.4526	0.7956
BGCN	0.9047	0.8913	0.6472	0.8235

Among the three traditional machine learning algorithms, SVM has the highest accuracy and F1, which are 88.67% and 0.7956, respectively. The F1 of CNN is similar to SVM, but the experimental effect of accuracy is 4% lower than that of SVM. The overall performance of LR is the worst among the three baselines. The experimental effect of the BGCN model is higher than that of the LR model in all evaluation metrics. Compared with CNN, the accuracy, precision, and F1 of the BGCN model are 19.18, 31.28, and 7.73% higher, respectively. To sum up, the accuracy, recall, and F1 of the BGCN model are higher than those of the other three machine learning algorithms, indicating the good performance of the BGCN model.

### Measures to Promote the Mental Health of College English Teachers

At present, the factors leading to the psychological pressure on college English teachers come from three aspects: society, colleges, and teachers themselves. Therefore, to promote the mental health of college English teachers, appropriate measures should be taken by society, colleges, and teachers themselves.

At first, reducing the psychological pressure of college English teachers and creating a good social atmosphere for teachers' personal career development are very important. To promote the mental health of college English teachers, it is necessary to guide the public opinion reasonably, improve the social status of college English teachers, and form a social atmosphere of respecting teachers and valuing education. Creating a healthy social environment, shaping a good image of teachers, promoting the professionalization of teachers, reducing the social burden and public pressure, establishing a social support system for teachers' mental health, and eliminating teachers' possible sense of psychological imbalance, all these aspects are very important (Hollett, 2021). At the same time, the government should continuously increase the investment in college English education, increase the number of teachers, improve the environment for college English teachers to work, study, and research, and support the innovation of college English teachers. While the government should also construct and improve a more perfect educational evaluation system, appropriately raise the income of college English teachers, and provide adequate care in housing, medical care, and other aspects to solve their worries.

Subsequently, colleges should help English teachers reduce their psychological pressure and create a good and harmonious internal working environment for their personal career development. All levels of colleges should understand the actual needs of English teachers and adopt appropriate incentive methods to improve teachers' psychological satisfaction and keep them in a good state of mind (Li, 2020). An important task of college management is to help teachers to solve the problems caused by psychological pressure. First, colleges should promote spiritual life and constantly improve the psychological endurance of English teachers, so that teachers can maintain psychological balance. At the same time, colleges should construct a good management mechanism, warm care for college English teachers, and establish an objective and fair teacher evaluation system to meet the motivation of achievement. In addition, colleges should improve the psychological stress relief mechanism of English teachers, and timely understand the psychological state of English teachers, so that most teachers can enjoy the work and receive mental health.

Finally, college English teachers should try their best to adjust themselves to psychological pressure and achieve personal career development. For college English teachers, external stressors cannot be changed for a while (Cheng and Lam, 2021). The key is to face pressure and psychological troubles calmly, relieve tension, regulate emotions, and avoid negative emotions from accumulating into diseases. College English teachers should have correct role cognition, learn to reasonably position themselves, objectively evaluate themselves, reasonably demand themselves, accept their own advantages and disadvantages, and establish appropriate career expectations. On the basis of careful analysis of their own professional characteristics, find and clarify the source of psychological pressure, try to solve their problems through different ways, and take a proactive way to deal with pressure. College English teachers must learn to relax their tension, pay attention to the combination of work and rest, and find an appropriate way for emotional venting. A strong will and good character can enable teachers to face all kinds of difficulties and setbacks to maintain an optimistic, cheerful, and calm state of mind and to calmly solve all unpleasant problems. This is a prerequisite for college English teachers to do a good job in teaching. An optimistic psychological state is a good medicine to promote teachers' physical and mental health. In addition, English teachers should change their ideas, pay attention to their own mental health status, learn psychological common sense, enhance mental health awareness and skills, and use psychological knowledge to constantly carry out self-liberation. Moreover, college English teachers should also strive to maintain harmonious interpersonal relationships with colleagues, undergraduates, and leaders, so as to further reduce various psychological pressures and achieve personal career development.

### Mental Health Management of English Teachers

Furthermore, we analyze the implementation path of improving college teachers' mental health. At present, the research on teachers' mental health service and management in colleges at home and abroad has made great progress. Colleges provide new welfare for teachers in practical work, which is a new form of welfare, namely “spiritual welfare,” in addition to traditional welfare such as salary, reward, and physical examination. To improve teachers' mental health, colleges need to implement a teachers' psychological assistance plan (Wang and Wang, 2011). On the one hand, it will help improve teachers' mental health literacy comprehensively. On the other hand, it will help colleges to form a warm and harmonious interpersonal environment. In this regard, education administrative departments should do a good job in planning and design, colleges should be responsible for organizing and implementing, and all departments within the college should cooperate to improve teachers' mental health literacy from the aspects of teachers' personal growth planning and guidance, family relationship handling, resolving occupational difficulties, and cultivate the original intention of education.

Psychological research shows that the need is the internal power of individual behavior and the basis and premise of emotion. Therefore, it is the premise of pertinently carrying out the work to clarify teachers' own growth needs. First, pay attention to the overall mental health of teachers. At present, mental health education in colleges has received wide attention, but the focus is mostly on undergraduates, and the mental health services for teachers have not been fully developed. With the intensification of competition and the change of lifestyle, the psychological problems of teachers derived from the pressure of life and work are increasing year by year. Therefore, it is necessary to bring the mental health service for teachers into the overall planning of mental health education in colleges. For example, regular psychological tests should be carried out for teachers, people with psychological abnormalities should be screened, and personal mental health files should be established. Then, attention should be paid to teachers' career depletion caused by their own development pressure. Through in-depth investigation and interviews with personnel departments and organizational departments, we can understand the personal needs and policy bottlenecks in the development of teachers and pay particular attention to the professional burnout caused by a mismatch of needs and unclear policies of teachers. Finally, the negative emotions caused by relationship conflicts should also be paid attention to. Therefore, colleges can regularly carry out college teachers' husband and wife relationship experience workshops, parent–child training camps, interpersonal relationship promotion counseling, teacher–undergraduate relationship discussion salon, etc. so that they can better improve their personal mental health level and feel the happiness brought by the improvement of mental health literacy.

Psychological research, counseling, and psychotherapy in developed countries appeared in the early twentieth century. At present, the mental health service system in developed countries has been relatively perfect, and the research field is also very extensive, which provides important reference for the psychological counseling service in China. Under the background of the new era, it is helpful to improve people's mental health literacy by providing standardized psychological services to different groups and expanding the recognition of psychological counseling. In recent years, with the increasing efforts of national mental health education, colleges have basically established a high-quality and complete psychological counseling team. In the future, colleges should appropriately open the psychological counseling service for teachers according to the actual situation of colleges. To avoid ethical issues, such as a double relationship in psychological counseling, colleges can purchase social services or introduce part-time social consultants to meet the psychological counseling needs of teachers. It is necessary to strengthen normative supervision, effect evaluation, and demand research so that teachers can feel the new experience brought by psychological counseling and the new changes brought by psychological care so that college teachers can fully understand the importance of psychological counseling and the necessity of seeking help in time, and then comprehensively improve their mental health literacy so as to promote their own development and social harmony.

Furthermore, English teachers should strive to improve their psychological quality. When threatened by pressure, English teachers should take the initiative to change from avoidance to solution, learn to treat problems objectively, and evaluate themselves objectively, so as to establish correct values, constantly improve their knowledge structure in the work environment, and establish new educational and professional views. As an English teacher shouldering the responsibility of cultivating cross-cultural communication talents, it is particularly important to maintain good psychological quality. Therefore, English teachers should not only improve their psychological quality but also actively overcome psychological barriers, constantly improve their knowledge structure, and keep up with the pace of English education reform so as to realize their own value.

## Conclusion

All kinds of influences of teachers' psychology on undergraduates' psychology play a very important role in undergraduates' physical and mental development. In today's development of quality education, the overall development of undergraduates is the embodiment of the quality of college education. With teachers who are physically and mentally healthy, there will be an all-round development of undergraduates. Therefore, colleges must focus on the mental health of teachers in teaching, especially during COVID-19. This study uses the data from the psychological test questionnaire of nearly 5,000 teachers in two colleges for research and analysis. We propose an individual recognition method for mental health crisis based on the BGCN model. Experimental results show that the accuracy of this model is 90.47%, precision is 89.13%, recall is 64.72%, and F1 is 0.8235, which are better than other machine learning models. The results show that the proposed method has a certain potential and is expected to be used in practice after a wider range of validation.

In the process of graph convolution, the embedding vector of the problem node in the last layer of the psychological test is still difficult to explain. This is a general problem based on the representation learning method. In the individual recognition of psychological crisis, interpretability is usually one of the important needs of the model. In the next research plan, a neural network model with high interpretability is proposed to realize the understanding of the embedding of question nodes, which is helpful to analyze the impact of various questions in the psychological test questionnaire on the individual recognition results of psychological crisis in more detail so as to further improve the practical value of this study.

## Data Availability Statement

The raw data supporting the conclusions of this article will be made available by the authors, without undue reservation.

## Ethics Statement

The studies involving human participants were reviewed and approved by the Ethics Committee of Heilongjiang University and Harbin Normal University. Written informed consent from the participants was not required to participate in this study in accordance with the national legislation and the institutional requirements.

## Author Contributions

YD contributed to the writing and analysis of the results. TW contributed to the methodology. Both authors contributed to the article and approved the submitted version.

## Funding

This work was supported by the Heilongjiang Project: Research on Etiquette Discourse Based on Ecological Linguistics under Grant No. 18YYC263, Educational Planning Project of Heilongjiang Province: Studies on Educational Space of Foreign Language Majors from the Perspective of New Literacy Education under Grant No. GJB1422037, and Application of Micro-Lessons in Basic Course Teaching of Engineering in Colleges and Universities under Grant No. SJGY20190193.

## Conflict of Interest

The authors declare that the research was conducted in the absence of any commercial or financial relationships that could be construed as a potential conflict of interest.

## Publisher's Note

All claims expressed in this article are solely those of the authors and do not necessarily represent those of their affiliated organizations, or those of the publisher, the editors and the reviewers. Any product that may be evaluated in this article, or claim that may be made by its manufacturer, is not guaranteed or endorsed by the publisher.
